# Designing a novel chimeric multi-epitope vaccine against *Burkholderia pseudomallei*, a causative agent of melioidosis

**DOI:** 10.3389/fmed.2022.945938

**Published:** 2022-10-18

**Authors:** Noorah Alsowayeh, Aqel Albutti

**Affiliations:** ^1^Department of Biology, College of Education (Majmaah), Majmaah University, Al Majmaah, Saudi Arabia; ^2^Department of Medical Biotechnology, College of Applied Medical Sciences, Qassim University, Buraydah, Saudi Arabia

**Keywords:** *Burkholderia pseudomallei*, melioidosis, immunoinformatics, molecular dynamics simulation, vaccine

## Abstract

*Burkholderia pseudomallei*, a gram-negative soil-dwelling bacterium, is primarily considered a causative agent of melioidosis infection in both animals and humans. Despite the severity of the disease, there is currently no licensed vaccine on the market. The development of an effective vaccine against *B. pseudomallei* could help prevent the spread of infection. The purpose of this study was to develop a multi-epitope-based vaccine against *B. pseudomallei* using advanced bacterial pan-genome analysis. A total of four proteins were prioritized for epitope prediction by using multiple subtractive proteomics filters. Following that, a multi-epitopes based chimeric vaccine construct was modeled and joined with an adjuvant to improve the potency of the designed vaccine construct. The structure of the construct was predicted and analyzed for flexibility. A population coverage analysis was performed to evaluate the broad-spectrum applicability of *B. pseudomallei*. The computed combined world population coverage was 99.74%. Molecular docking analysis was applied further to evaluate the binding efficacy of the designed vaccine construct with the human toll-like receptors-5 (TLR-5). Furthermore, the dynamic behavior and stability of the docked complexes were investigated using molecular dynamics simulation, and the binding free energy determined for Vaccine-TLR-5 was delta total −168.3588. The docking result revealed that the vaccine construct may elicit a suitable immunological response within the host body. Hence, we believe that the designed *in-silico* vaccine could be helpful for experimentalists in the formulation of a highly effective vaccine for *B. pseudomallei*.

## Introduction

*Burkholderia pseudomallei*, a member of the family *Burkholderiaceae*, is a rod-shaped, gram-negative, motile, and multitrichous flagella bacterium typically of 1–5 μm long and diameter of 0.5–1.0 μm (1). Melioidosis, often known as Whitmore's infection, is caused by *B. pseudomallei* and is characterized by

high mortality and morbidity rates (2). The infection usually leads to abscess formation and sepsis. This pathogen infects both animals and humans when they come into contact with contaminated soil (3). The bacteria can be spread from an infected person to a non-infected person and can be acquired in the hospital environment as well. Despite the rarity of zoonotic transmission and animal-to-animal infection transfer, a wide variety of species have contracted the disease (4). The bacterium *B. pseudomallei* is thought to have a role in horizontal gene transfer (4). Melioidosis infection is prevalent in tropical regions while the main endemic regions include Southeast Asia and northern Australia (5, 6). Every year, around 165,000 cases of Melioidosis are reported worldwide, with 89,000 deaths (7). A high mortality rate of 42.6% was estimated in Thailand from 1997 to 2006 (6). By 2018, 30–35% of infections in Thailand's hospitals resulted in deaths (8). The bacterium is regarded as a biological weapon, and it has been linked to diabetes, chronic lung and renal diseases, malignancies, and heart diseases. Earlier diagnosis of the disease can decrease the mortality rate. Melioidosis chronic infection is frequently associated with the risk factors indicated above (9). The remarkable *B. pseudomallei*'s adaptative characteristics allow the bacteria to survive in the host body and neutralize host immune responses (2). As this infection is associated with many clinical manifestations, its diagnosis is considered difficult (10). The incubation period also varies but usually, it is 1–21 days (11). In case of infection, the infected person's blood test generally has elevated liver enzymes, higher levels of urea and creatinine, low blood glucose levels, and fewer white blood cell counts (2). Most of the infected individuals have pneumonia. The pathogen affects internal organs like the spleen, kidney, and liver (12). Toll-like receptors (TLRs) play an important role in inducing innate immunity. Infected patients showed increased expression of TLRs (13).

Early antimicrobial therapy against the infection proved effective in lowering the mortality rate in endemic regions (11). The pathogen shows resistance to several antibiotics, including cephalosporins, imipenem, meropenem, tetracyclines, sulphonamides, etc. The effectiveness of antimicrobial therapy depends upon the duration of the treatment, which in normal cases is up to 6 months (14). A licensed vaccine for Melioidosis has not been not reported to date, but recent studies confirmed the pre-clinical trials of *B. pseudomallei* vaccine in animal models (15). Several strategies for vaccine development against *Burkholderia* have been explored, but none of them induce sterilizing immunity for a long time. Some promising live attenuated and subunit vaccines against *Burkholderia* have been proposed. Many of the studies revealed short-term immune protection against the pathogen in animal models (15). The experimental vaccine development can be assisted with computational vaccine approaches that use genomic information of bacterial pathogens to predict novel antigenic peptides. The peptide vaccines are easy to produce and much cheaper compared to whole or subunit-based vaccines. This study highlights several vaccine targets against the pathogen using immunoinformatic approaches. A multi-epitope peptide vaccine is constructed along with MALP-2 (macrophage activating lipoprotein 2) adjuvant. Computational analysis of the vaccine highlighted its good ability to induce protective responses against the pathogen. This strategy is considered simple and cost effective and the findings might be useful for multi-epitope peptide vaccine development against *B. pseudomallei*.

The multi-epitopes vaccine (MEVC) construct has many advantages over the single peptide-based vaccine. MEVC elicits both humoral and cellular immune responses because it comprises both B and B-cell derived T-cell epitopes (16, 17). Other advantages of MEVC include probable antigenicity, less toxicity, non-allergenicity, and good water-soluble properties (18). The MEVC in the present study comprises multiple epitopes which were prioritized from vaccine targets. The vaccine proteins were filtered using pan-genome analysis and reverse vaccinology techniques. Extracellular, periplasmic, and outer membrane proteins were shortlisted for epitope mapping. B-cell derived T-cell epitopes were predicted and potential epitopes were used to design a vaccine construct which further underwent different computational analyses. Toll-like receptor 5 (TLR5), shows high expression in Melioidosis (15) and is a receptor for bacterial flagellin. This receptor was used for molecular docking advanced by molecular dynamics simulation and binding free energies.

## Methodology

### Core proteome retrieval

**Figure 1:**
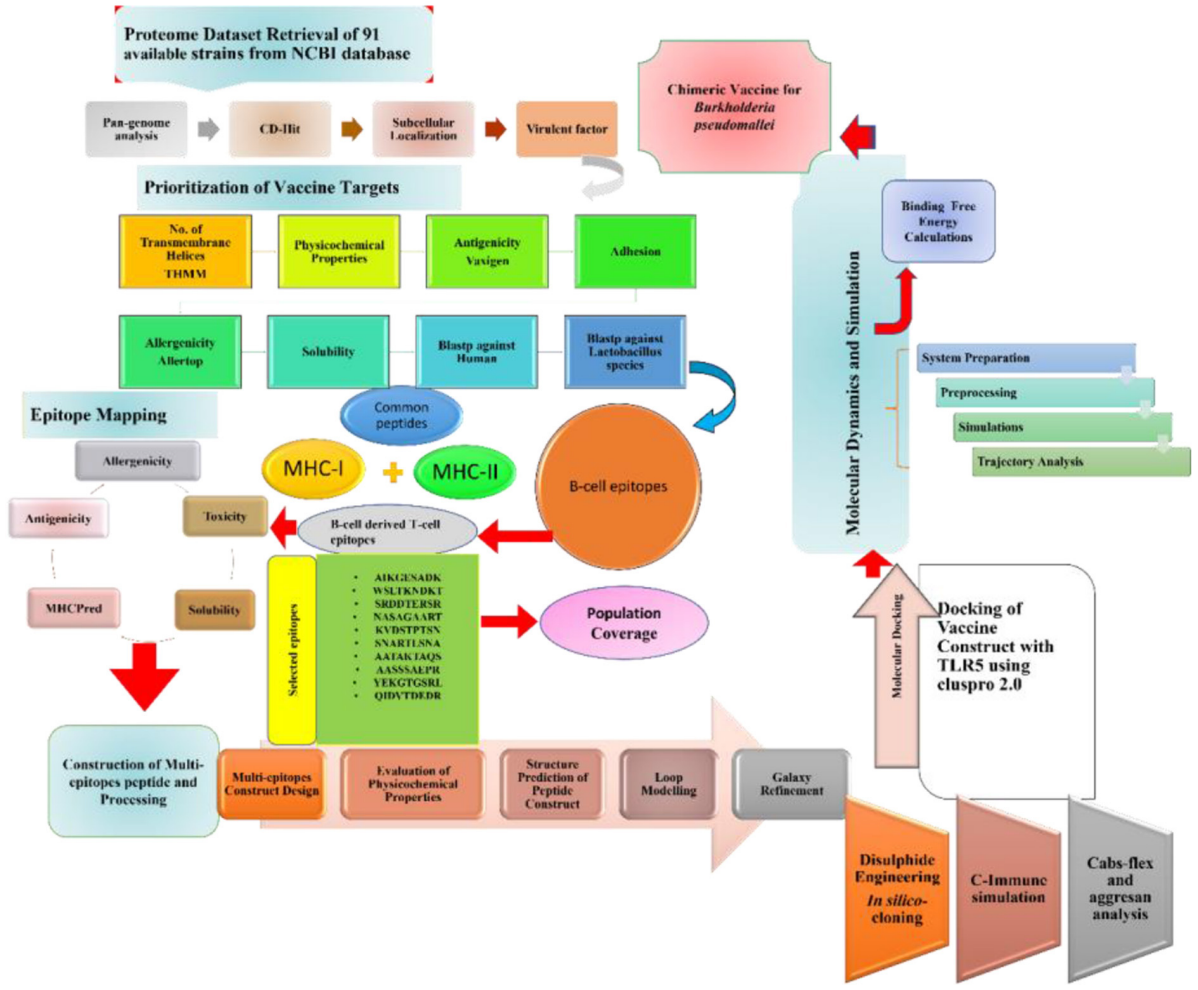
Schematic diagram of the methodology and steps followed in this study.

Completely sequenced 91 proteomes of *B. pseudomallei* were obtained from the National Center for Biotechnology Information (NCBI) (19) followed by the retrieval of the core proteomes of the species by applying the bacterial pan genome analysis tool (BPGA) (19). CD-HIT was then used to extract non-redundant sequences at a 90% threshold. CD-HIT is a cluster database used to analyze the proteomes by removing similar sequences. PSORTB v 3.0 (20) was used for subcellular localization. Subcellular localization refers to the protein localization prediction in *B. pseudomallei*. The outer membrane, extracellular, and periplasmic proteins were chosen for epitope mapping leading to multi-epitope vaccine designing. The protein sequences obtained includes 39 extracellular, 53 periplasmic, and 55 outer membranes which were then subjected to further analysis as presented in Figure 1.

### Identification of potential vaccine candidates

All the extracellular, periplasmic, and outer membrane proteins were checked for transmembrane helices using TMHMM 2.0 (https://services.healthtech.dtu.dk/service.php?TMHMM-2.0). Physiochemical properties like molecular weight, theoretical index, instability index, and GRAVY (hydrophilicity check) for proteins with transmembrane helices < 2 were checked by using the Protparam tool (https://web.expasy.org/protparam/). The molecular weight of the proteins should be <100 kDa as they can be easily purified from the cell, and their instability index should be <45. Further, antigenicity and allergenicity of the proteins were checked *via* Vaxijen v2.0 (21) and AllerTOP v2.0, respectively (22). The Innovagen server was used to shortlist the soluble proteins. The adhesion probability of the proteins was evaluated using Vaxign webserver (23). Finally, BLASTp was performed on shortlisted proteins against human and lactobacillus species to avoid auto-immune responses and to restrict accidental inhibition of the probiotic bacteria, respectively. The sequence identity and bit score used in this process were ≤30 % and 100, respectively.

### Epitope prediction and prioritization

All the protein sequences meeting the criteria of being a potential candidate and shortlisted from all the checks were then used for epitope mapping utilizing the Immune Epitope Database and Analysis Resource (IEDB) (24). The proteins subjected to epitope mapping were antigenic, non-allergen, soluble, and adhesion probability > 0.5. B-cell epitope mapping was done using the B-cell epitope prediction tool of IEDB. The predicted B-cell epitopes were then used as input sequences for MHC-II (major histocompatibility complex II) binding epitopes prediction, and the anticipated MHC-II binding epitopes were used to predict MHC-I binding epitopes. A complete reference set of alleles were selected for T-cell epitope (MHC-I & MHC-II binding) mapping. The B-cell derived T-cell epitopes were further analyzed for their binding affinity by MHCpred v2.0 (http://www.ddg-pharmfac.net/mhcpred/MHCPred/). Epitopes with IC50 <100 nM were considered good binders and further scrutinized for antigenicity, allergenicity, toxicity, and solubility. Only epitopes that were antigens, non-allergen, non-toxic, and soluble were used to design a multi-epitope vaccine construct.

### Population coverage analysis

Population coverage analysis of the designed vaccine was evaluated using the Population coverage tool of IEDB. The alleles which cover most of the world population were used for the analysis.

### Multi-epitopes vaccine designing and 3D modeling

Shortlisted epitopes were linked together to design a vaccine construct. GPGPG linkers were used to link epitope to epitope while EAAAK linker was used to connect epitopes peptide with an adjuvant. The adjuvant molecule was used to boost vaccine immune responses. Here, we used four different adjuvants resulting in four different constructs which were further analyzed for physiochemical properties, antigenicity, allergenicity, adhesion probability, and secondary structures. Construct with Cholera toxin-B (CTB) adjuvant met the criteria to be considered best amongst the four. MALP-2 (GNNDESNISFKEK) was added to the construct, EAAAK linker was used to link the last epitope and MALP2 sequence. MALP-2 (macrophage activating lipoprotein) is a toll-like receptor (TLR) agonist mostly used to treat certain infections and also as an adjuvant for a vaccine. Physiochemical properties like molecular weight, hydrophilicity, theoretical, and instability index of the final vaccine construct were evaluated by the ProtParam tool (25). We predicted the 3D structure modeling of the vaccine using the Scratch predictor tool (25). The vaccine model was analyzed for the presence of the loops and subjected to loop modeling via Galaxy loop followed by refinement with Galaxy refine v 2.0 tool, respectively (26).

### Cabs-flex analysis

The cabs-flex analysis is a computational approach to investigating the structural flexibility of the protein. The multi-epitopes peptide vaccine was subjected to coarse-grind simulations using Cabs-flex 2.0 (27). The analysis was run for 50 cycles and an RNG seed of 8335. The temperature range, weight of global side chain restraint, and the weight of global C-alpha restraints used in the analysis were 1.40, 1.0, and 1.0, respectively. This analysis proceeded by following the identification of aggregation-prone sites in protein sequence (vaccine) by AGGRESCAN 3D server v2.0 (http://bioinf.uab.es/aggrescan/).

### Disulfide engineering and *in-silico cloning*

Disulfide by Design 2.0 was used to perform disulfide engineering of the vaccine model. It is usually done to identify unstable residues being mutated into cysteine residues narrating the induction of disulfide bonds making the structure stable (27).

Java Codon Adaptation Tool (JCat) (28) was used for the codon optimization of the vaccine construct. Through JCat, the vaccine protein sequence is translated into nucleotide sequence. Additionally, the CAI (codon adaptation index) value and GC content were determined, enabling *in-silico* cloning *via* Snapgene (29). The *in silico* cloning was done in vector pET-28a (+).

### Computational immune simulation

A computational immune simulation is an approach to analyzing the vaccine construct for its immunogenicity and ability to induce immunity. This analysis was done through the C-ImmSim webserver (30). The webserver uses machine learning techniques while predicting the immune cells and antigen interaction.

### Molecular docking

Toll-like receptor-5 (TLR-5) and vaccine model (ligand) were subjected to molecular docking using an online docking tool ClusPro 2.0 (31). Several docked solutions were generated and ranked based on binding energy. The solution with the lowest binding energy was chosen for further investigation. The interactions between the receptor and the vaccine were visualized through UCSF Chimera v 1.15, PDBsum (http://www.ebi.ac.uk/thornton-srv/databases/pdbsum/Generate.html) and Discovery Studio v2021.

### Molecular dynamics simulation

Molecular dynamics simulation was performed for the docked complex of the vaccine model and TLR5 *via* (Assisted Model Building with Energy Refinement tool) AMBER 20 software. The simulation was conducted for 50 ns to understand the dynamic behavior in an aqueous solution. The force field “ff14SB” was used to generate parameters for both the vaccine and the TLR5, followed by incorporation of complex into TIP3P water box by maintaining 12 Å padding distance. The Na^+^ ions were added to neutralize the system. The carbon alpha atoms, non-heavy atoms, hydrogen atoms, and solvation box energies were minimized for 500, 1,000, 300, and 1,000 steps, respectively. Langevin dynamics were applied to maintain the system's temperature by the execution of system heating to 300 K for 20ps. The SHAKE algorithm was applied to constraint hydrogen bonds. The production run trajectories were analyzed by using the AMBER CPPTRAJ module. Binding free energies were predicted using the MMPBSA.py module (https://ambermd.org/tutorials/advanced/tutorial3/py_script/section4.htm).

## Results

### Retrieval of core proteome and determining the vaccine candidates

**Figure 2:**
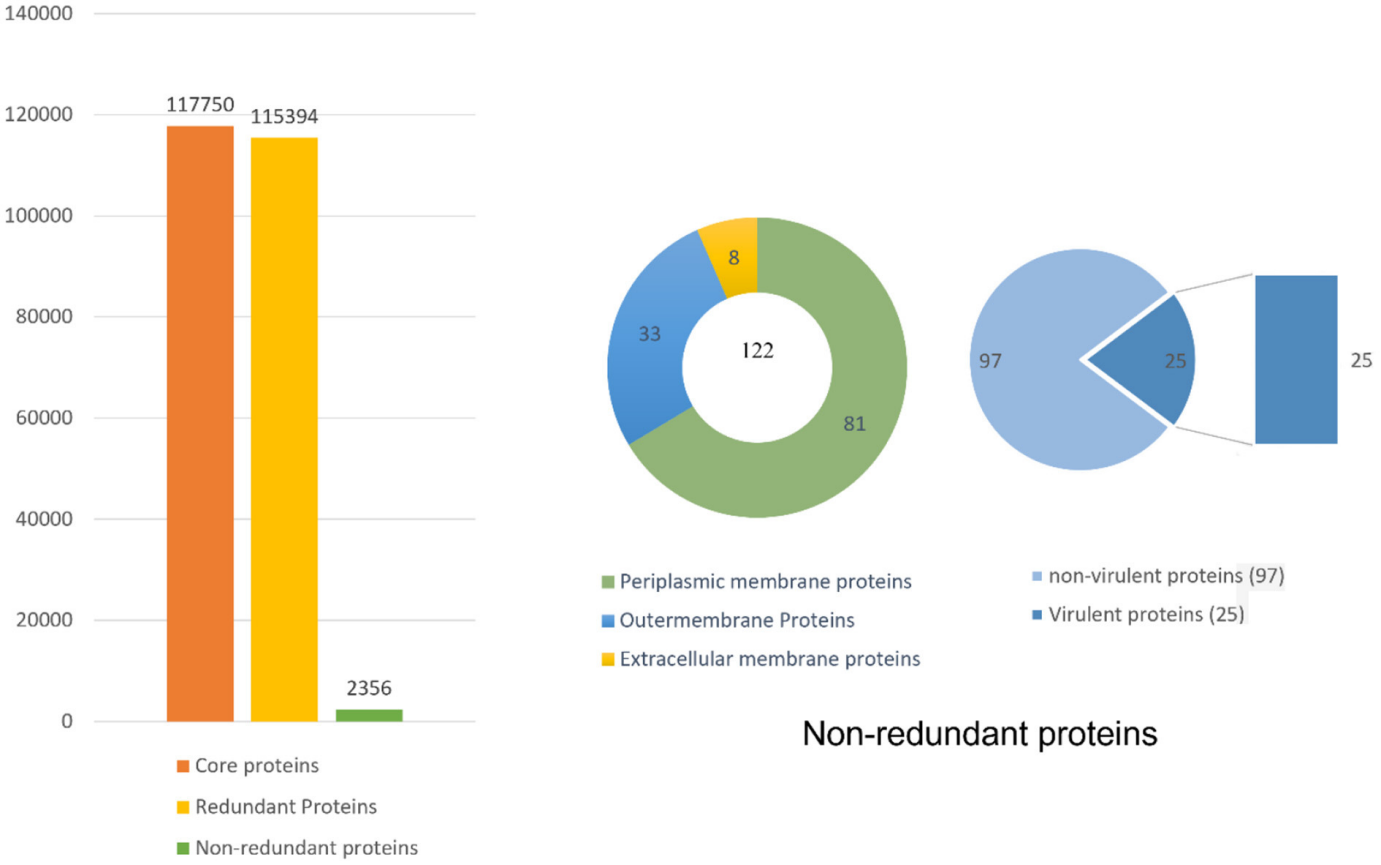
Complete detail of the total retrieved and shortlisted proteins.

Pan-genome analysis was conducted for 91 completely sequenced proteomes of *B. pseudomallei* to get core proteome (19). The total number of the core proteins obtained were 310,128, which were analyzed through the CD-HIT analysis. The non-redundant sequences obtained were 3,647 while all the duplicate sequences were removed. As only extracellular, periplasmic, and outer membrane proteins were of interest so the subcellular localization of those 3,647 sequences was done by PSORTb. Sequences obtained were 39, 56, and 53 in number for extracellular, periplasmic, and outer membrane proteins, respectively (Figure 2).

The bacterial virulence factor was determined using the virulence factor database (VFDB). The protein sequences with a sequence identity of more than 30% and a bit score >100 were considered the best vaccine targets. Virulent proteins numbered 17 in the extracellular group, 18 in the periplasmic group, and 12 in the outer membrane group. The sequences were further analyzed for the presence of transmembrane helices that should be 0 or 1. The number of transmembrane helices was 0 for extracellular proteins, except for one. In the periplasmic group, only one had transmembrane helices out of 18 proteins while the remaining were lacking the transmembrane helices. Out of the 12 outer membrane proteins, only three (core/2096/1/Org1 Gene676, core/4074/1/Org1 Gene4523, and core/4761/73/Org73 Gene5861) had transmembrane helices, while the rest had 0. In all three groups, the proteins with no transmembrane helices were subjected to physicochemical evaluation. The parameters evaluated were molecular weight, theoretical index, GRAVY (Grand average of hydropathy), and instability index. Molecular weight was <100 kDa, while the GRAVY calculated was negative and the proteins having an instability index >40 were considered unstable proteins. The instability index of 15 extracellular proteins, 13 periplasmic, and nine outer membrane proteins were stable and within the range of 40. All these stable proteins were subjected to antigenicity (threshold 0.5), allergenicity, solubility, and adhesion stability (threshold 0.5) checks. All the non-antigenic and allergen proteins were discarded including eight extracellular, four periplasmic, and two outer membrane proteins. Similarly, the poorly soluble and the proteins having adhesion probability <0.5 were discarded, and only antigenic, non-allergens, soluble, and having adhesion probability were shortlisted as potential vaccine candidates. Four proteins were filtered out of which three (core/7319/1/Org1_Gene2732, core/7521/1/Org1_Gene303, and core/8978/1/Org1_Gene1539) were from the extracellular group and only 1 (core/3042/1/Org1_Gene5247) was a periplasmic protein as listed in Table 1. The BLASTp against human and lactobacillus species was also run for the shortlisted proteins that reported no significant similarity between the proteins and human/probiotic proteomes. The core/7319/1/Org1_Gene2732 protein is a type VI secretion system involved in the transportation of effector molecules from a bacterial cell to the target cell, core/7521/1/Org1_Gene303 is an Hcp family type VI secretion system effector which is present in the outer membrane and play a role in pathogenicity. The core/8978/1/Org1_Gene1539 is a flagellar biosynthesis anti-sigma factor FlgM which acts as a negative regulator in synthesizing flagellin and core/3042/1/Org1_Gene5247 is D-alanyl-D-alanine endopeptidase (32–34).

**Table 1:** Properties for the potential vaccine candidates.

**Gene Id**	**Name**	**Function**	**Length**	**VFDB**	**T.H**	**M.W**	**T. pI**	**I. I**	**GRAVY**	**Antigenicity**	**Allergenicity**	**Solubility**	**Adhesion**
core/7319/1/Org1_ Gene2732	Type VI secretion system	Transport the effector molecules from bacterial cell to target	167	46.71	0	18.33	6.43	37.04	−0.472	1.0872	Non-allergen	soluble	0.708
core/7521/1/Org1_ Gene303	Hcp family type VI secretion system effector	Play role in pathogenicity	161	64.38	0	17.54	5.57	38.01	−0.501	1.146	Non-allergen	soluble	0.586
core/8978/1/Org1_ Gene1539	Flagellar biosynthesis anti-sigma factor FlgM	Negative regulator in synthesizing flagellin	114	60.53	0	11.11	8.19	20.6	−0.239	0.728	Non-allergen	soluble	0.665
core/3042/1/Org1_ Gene5247	D-alanyl-D-alanine endopeptidase, putative	Hydrolyzing the peptidoglycan	373	46.42	1	39.91	9.99	35.83	−0.231	0.534	Non-allergen	soluble	0.601

### Epitope prediction and prioritization

In epitope mapping, T-cell epitopes were predicted from B-cell epitopes for eliciting both humoral and cellular immune responses. Using the shortlisted proteins as input sequences, the B-cell derived T-cell epitopes were predicted by IEDB. The B-cell epitope prediction tool of IEDB was used to predict potential peptides acting as B-cell epitopes. Peptides having a value ≥0.5 (threshold) were considered as B-cell epitopes. Thirteen peptides were predicted as B-cell epitopes. A total of 78 T-cell MHC class II binding epitopes were predicted from the B-cell epitopes. These epitopes were then used to predict the MHC class I binding epitopes on T-cells, and 120 of these epitopes with lengths of 9 or 10 residues were selected based on their percentile rank. The compete reference set of alleles was selected as a parameter for MHC class II and I binding epitopes prediction.

The predicted epitopes were prioritized using different parameters. First, they were subjected to MHCPred to select epitopes with IC50 values <100 nM to confirm the epitopes as good binders. With the help of this analysis, 64 epitopes were chosen and further examined by VaxiJen for antigenicity; only 52 epitopes were antigenic, of which 24 were non-allergen. All the epitopes were non-toxins. Five epitopes were predicted by Innovagen to be poorly soluble and were deleted while overlapping ones were also removed. Finally, 10 epitopes listed in Table 2 were chosen for a downward analysis.

**Table 2:** Predicted B cell derived T cell epitopes with various checks.

**Protein IDs**	**Protein names**	**B cell epitopes**	**MHC II binding epitopes**	**MHC I binding epitopes**	**MHC Pred**	**IC50 value**	**Antigenicity**	**Allergenicity**	**Toxicity**	**Solubility**
core/7319/1/Org1_ Gene2732	Type VI secretion system	PAIKGESADKDHE	AIKGESADKDH	AIKGESADK	AIKGESADK	75.16	1.7824	Non-allergen	Non-toxin	Soluble
		WKQTQQKIGGNQGGNTQGAWSLTKNDKTYA	WSLTKNDKTYA	WSLTKNDKTY	WSLTKNDKT	74.13	0.822	Non-allergen	Non-toxin	Soluble
core/7521/1/Org1_ Gene303	Hcp family type VI secretion system effector	RPSGSRDDTERSRE	SGSRDDTERSR	GSRDDTERSR	SRDDTERSR	10.48	1.6539	Non-allergen	Non-toxin	Soluble
core/8978/1/Org1_ Gene1539	Flagellar biosynthesis anti-sigma factor FlgM	MKVDSTPTSNARTLSNASAGAARTQAGQPAAAQTPAGAAGAPTGGDANV	LSNASAGAART	NASAGAART	NASAGAART	9.31	1.6869	Non-allergen	Non-toxin	Soluble
		MKVDSTPTSNARTLSNASAGAARTQAGQPAAAQTPAGAAGAPTGGDANV	MKVDSTPTSNA	KVDSTPTSNA	KVDSTPTSN	23.82	1.2218	Non-allergen	Non-toxin	Soluble
		MKVDSTPTSNARTLSNASAGAARTQAGQPAAAQTPAGAAGAPTGGDANV	TSNARTLSNAS	TSNARTLSNA	SNARTLSNA	9.46	0.5532	Non-allergen	Non-toxin	Soluble
core/3042/1/Org1_ Gene5247	D-alanyl-D-alanine endopeptidase, putative	VAPADAFAATAKTAQSAKGKKSAAKK	AFAATAKTAQS	AATAKTAQS	AATAKTAQS	7.8	1.153	Non-allergen	Non-toxin	Soluble
		LRAASSSAEPRAKGAR	LRAASSSAEPR	AASSSAEPR	AASSSAEPR	37.85	1.52	Non-allergen	Non-toxin	Soluble
		KSPLTDQIDVTDEDRDYEKGTGSRL	RDYEKGTGSRL	YEKGTGSRL	YEKGTGSRL	4.7	1.9551	Non-allergen	Non-toxin	Soluble
		KSPLTDQIDVTDEDRDYEKGTGSRL	QIDVTDEDRDY	QIDVTDEDR	QIDVTDEDR	5.36	1.0121	Non-allergen	Non-toxin	Soluble

### Population coverage

**Figure 3:**
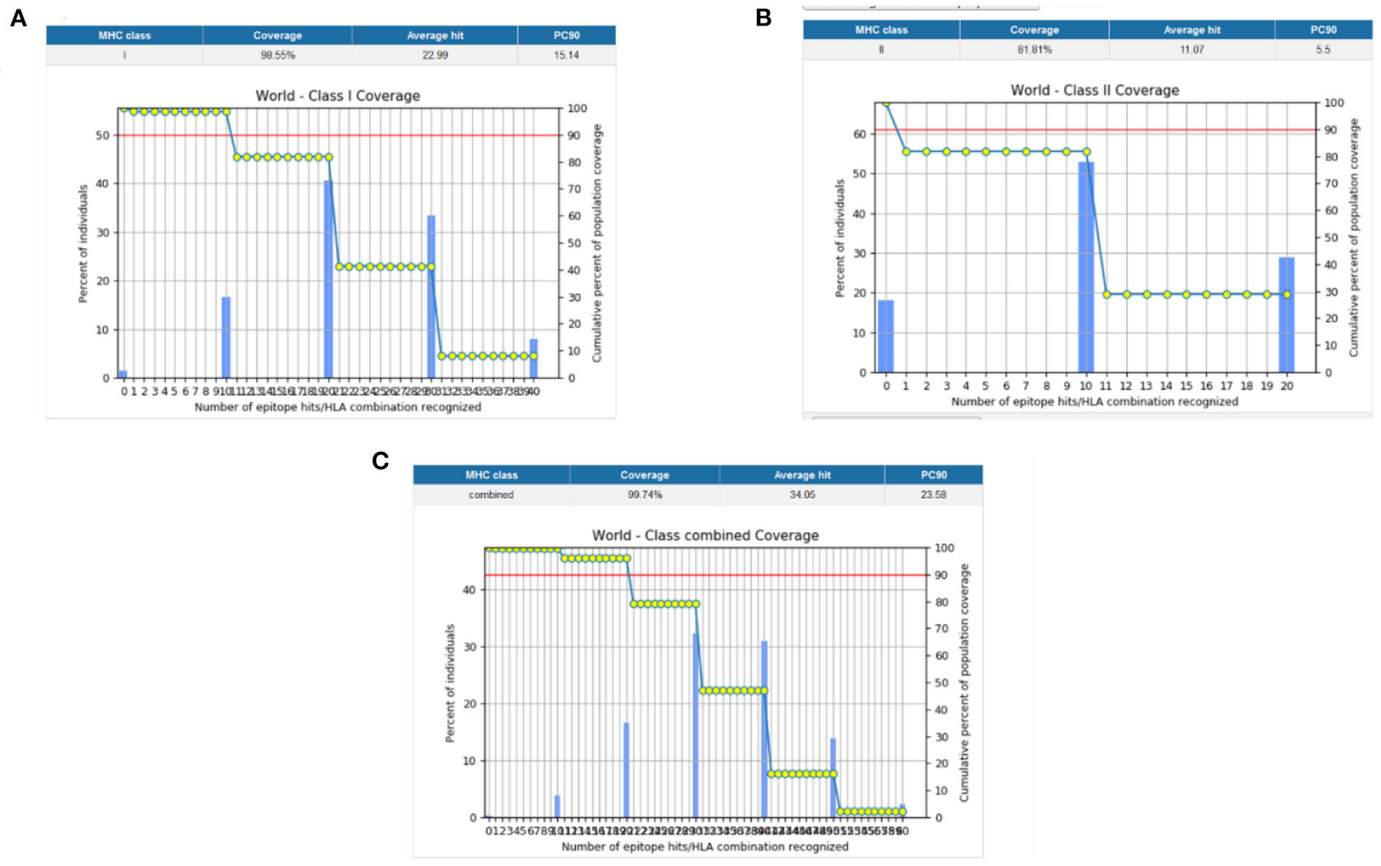
**(A)** MHC class I world population coverage **(B)** MHC class II world population coverage **(C)** MHC class I and II combined world population coverage.

**Figure 4:**
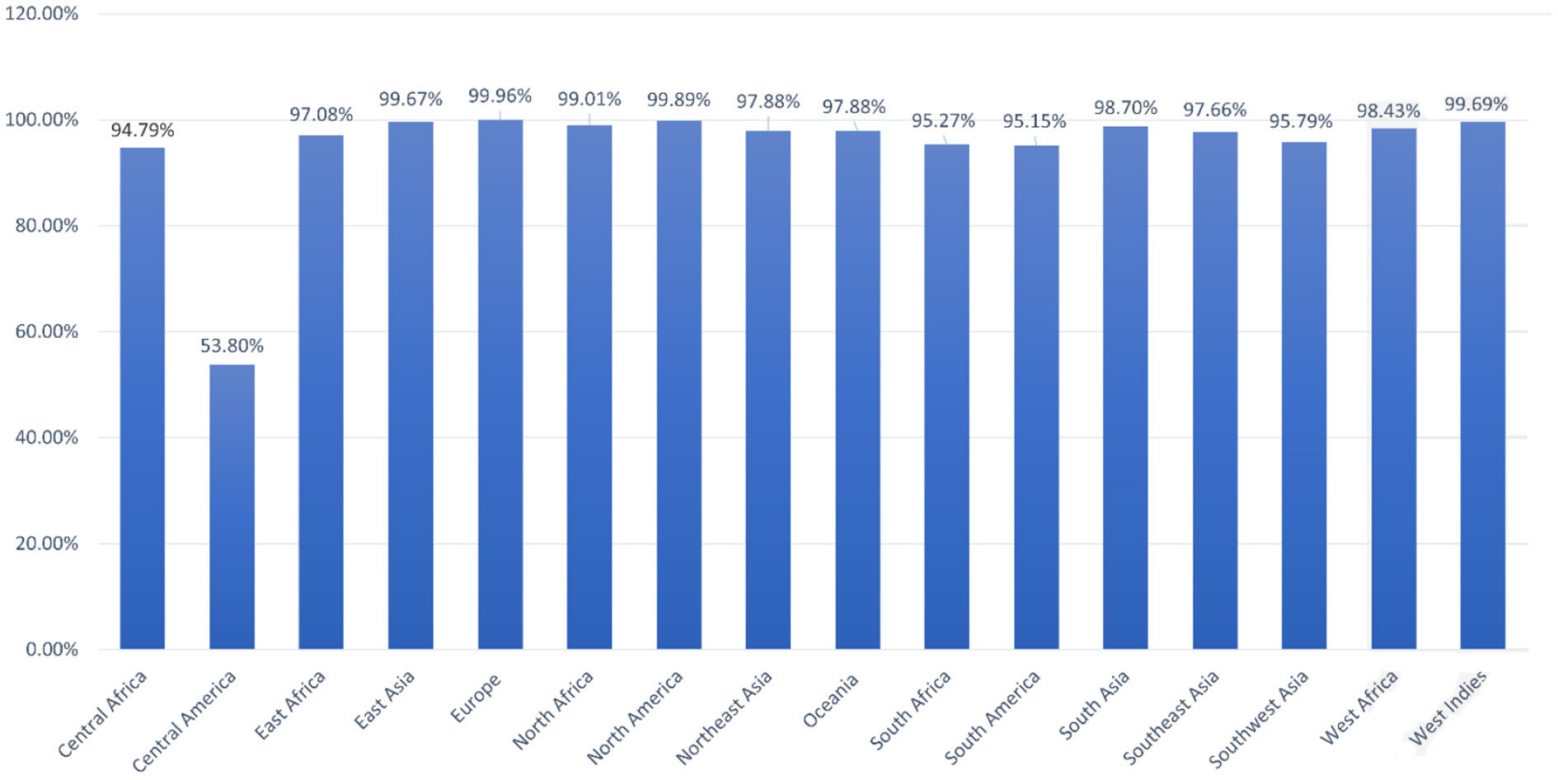
World population coverage of the designed vaccine construct.

Population coverage analysis by IEDB was performed for the shortlisted epitopes to estimate their binding probability to MHC molecules covering the world population (35). World population coverage calculated for MHC class I was 98.55% (Figure 3A) and for MHC class II was 81.81% (Figure 3B). The combined population coverage for MHC molecules was 99.74% as in Figure 3C. MHC class combined world population coverage was also computed region-wise, as shown in Figure 4.

### Designing the vaccine constructs and 3D modeling

**Figure 5:**
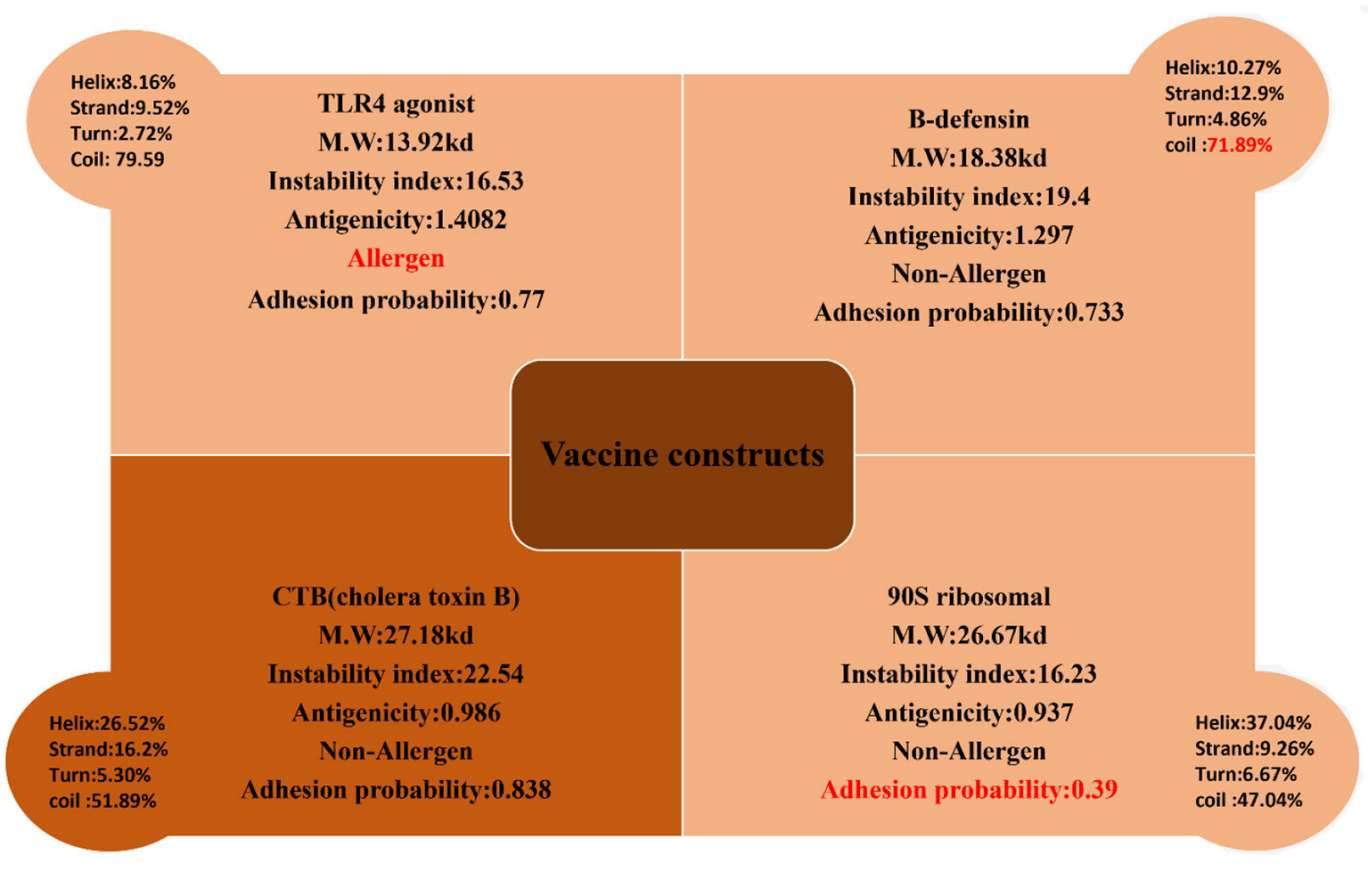
Four constructs with different adjuvant were analyzed for their physiochemical properties and secondary structure parameters. One construct with a TLR4 agonist was an allergen, the construct with B-defensin had a higher number of coils depicting the unstable structure, and the construct with 90S ribosomal had a very low adhesion probability; these were discarded. The construct with CTB met the criteria of a potential vaccine.

The 10 shortlisted epitopes that were antigenic, non-allergen, non-toxic, and soluble were considered as potential epitopes to be a part of the vaccine construct. Epitopes were chosen using GPGPG linkers. We designed four constructs using four different adjuvants like TLR-4 agonist, B-defensin, Cholera B toxin (CTB), and 50 s ribosomal adjuvant. The rationale for using different adjuvant molecules was to check which adjuvants show the best compatibility with the designed vaccine molecule and generate strong and protective immune responses. The designed constructs were then evaluated for their physiochemical properties, antigenicity, allergenicity, adhesion probability, and prediction of secondary structure on four parameters (Figure 5). The construct with the adjuvant TLR4 agonist was marked as allergen while the construct with adjuvant 50S ribosomal had an adhesion probability of 0.3 which was less than threshold 0.5 so they both were eliminated. The remaining vaccine constructs one with the adjuvant B-defensin and the other with Cholera B toxin were compared. The vaccine construct with the adjuvant CTB was shortlisted based on its secondary structure being stable. We linked MALP-2 (macrophage activating lipoprotein 2) with construct *via* the EAAAK linker at the end presumed to be involved in enhancing the antigenicity of the construct. The CTB vaccine construct along with MALP 2 was again evaluated for physiochemical properties thus having a molecular weight of 29.01 kDa, theoretical index of 8.92, −0.65 GRAVY, and stable with a 23.28 instability index. It was non-allergen and antigen with antigenicity of 1.0059.

**Figure 6:**
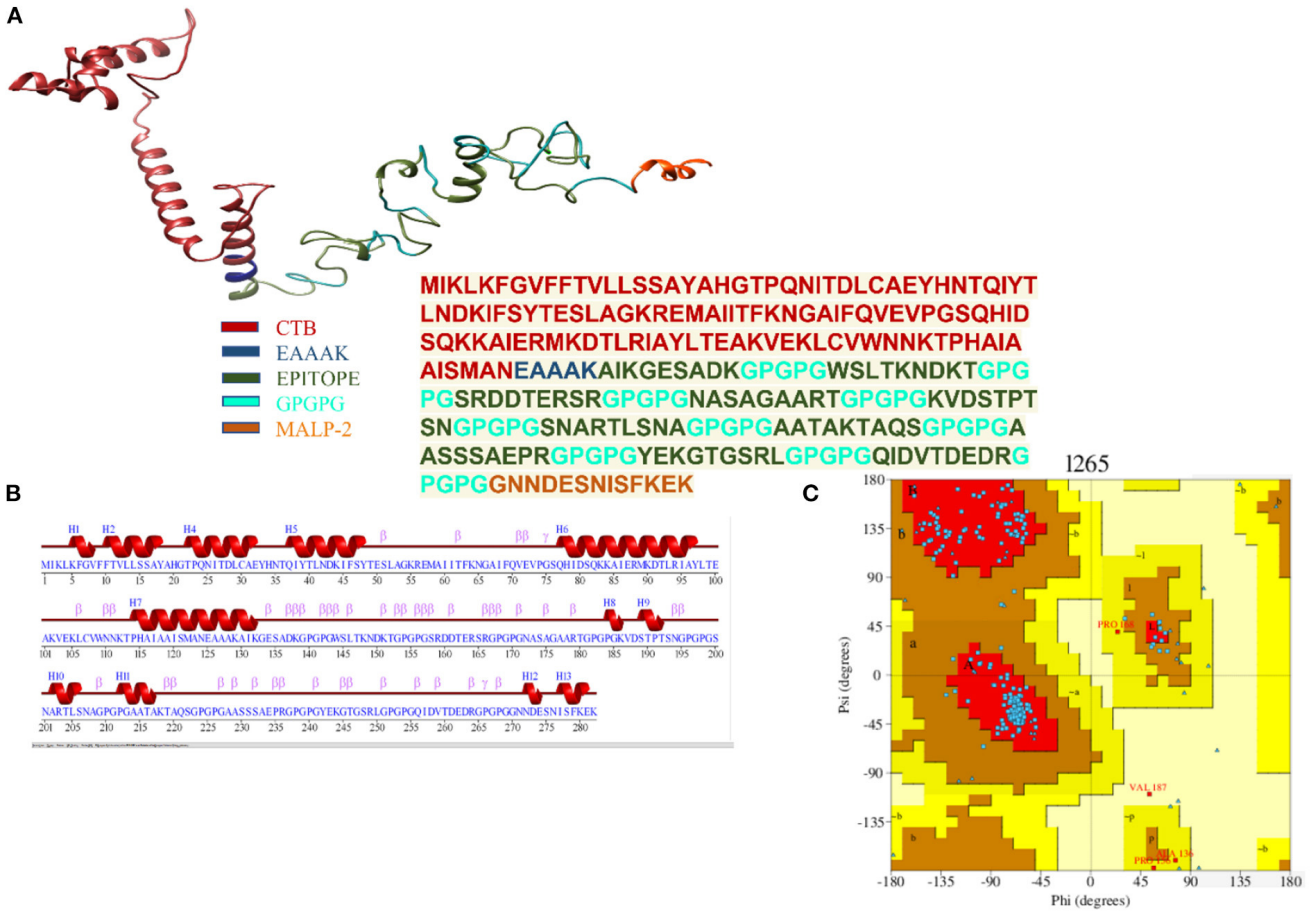
**(A)** Depiction of the details of 3D model of the multi-epitope vaccine with peptide sequence for the vaccine construct. **(B)** Secondary structure for the multi-epitope vaccine. **(C)** Ramachandran plot showing 92.6% of the residue in most favorable region.

The finalized vaccine construct was modeled into a 3D structure (Figure 6A) using the Scratch predictor and visualized by UCSF chimera which highlights the loop regions in the structure. The structure was loop modeled by the Galaxy loop of the Galaxy web and was refined by the Galaxy refine of the Galaxy web. The top 10 galaxy refined models along with their structural information are shown in Table 3. 2D structure analysis and structural validation were done through PDBSum Generate tool (35). Figure 6B is highlighting the secondary structure elements of the vaccine model having 13 helices, 6 helix-helix, 46 beta turns, and 3 gamma turns. Ramachandran plot as shown in Figure 6C confirmed the presence of 199 residues in the Rama favored region, 14 were in additional allowed and 1 was in disallowed region, hence validating a good model for the vaccine.

**Table 3:** Galaxy refined models with their properties.

**Model**	**GDT-HA**	**RMSD**	**MolProb**	**Clash score**	**Poor rotamers**	**Rama favored**
Initial	1	0	2.992	56.3	1.4	85.4
Model 1	0.9459	0.436	1.832	9.7	0.9	95.4
Model 2	0.9592	0.398	1.914	10.7	0.9	94.6
Model 3	0.9477	0.421	1.86	10.4	0.5	95.4
Model 4	0.9486	0.417	1.877	9.7	0.9	94.6
Model 5	0.9504	0.406	1.883	10.4	0.9	95

### Cabs-flex analysis

**Figure 7:**
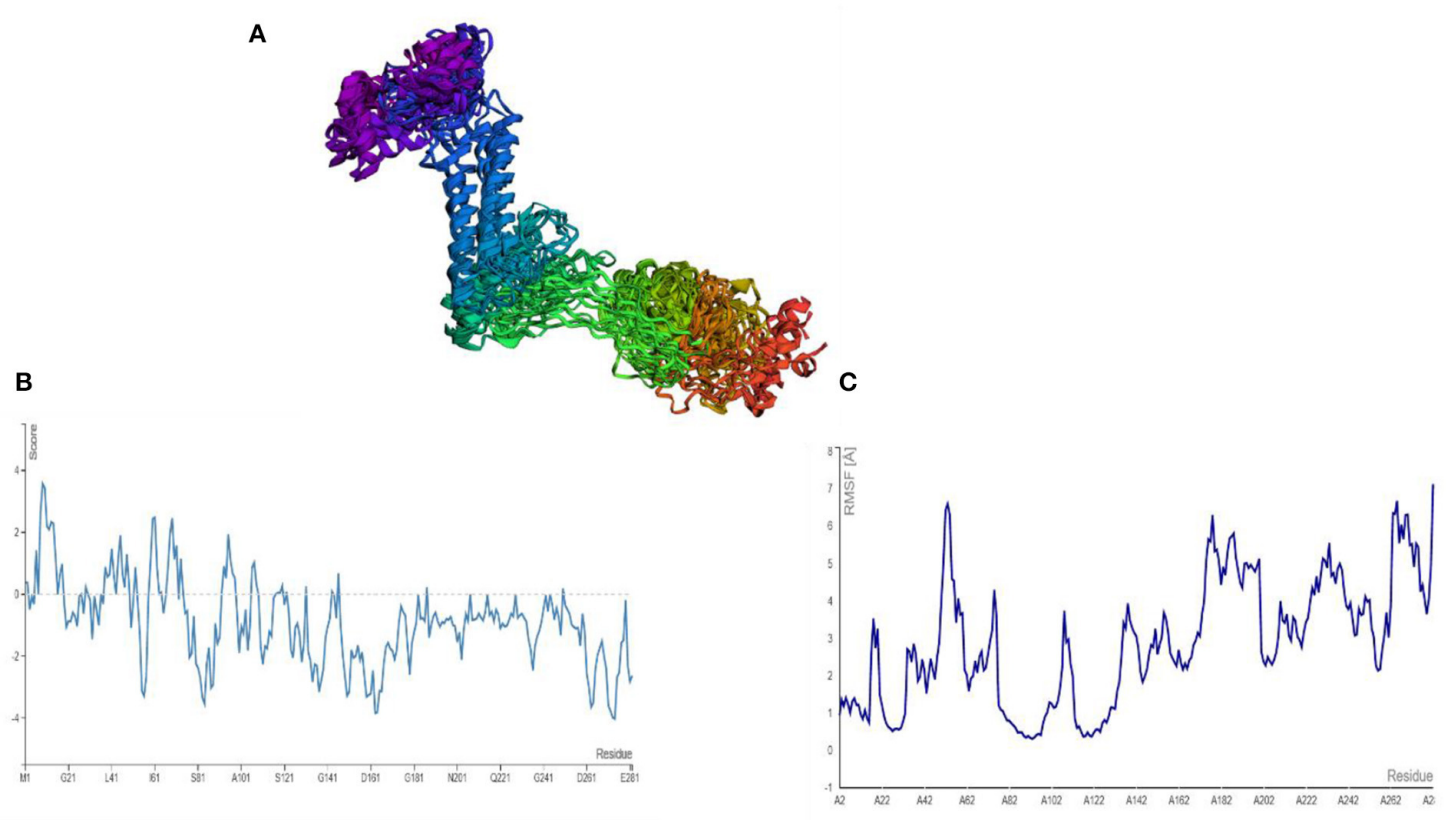
**(A)** Predicted models after a cabs-flex analysis. **(B)** AGGRESCAM plot which depicts that the residues with a score above zero are aggregation prone residues. **(C)** Cabs-flex fluctuations plot, fluctuations calculated as RSMF.

AGGRESCAN analysis was performed before a cabs-flex analysis (cabs-flex obtained model is shown in Figure 7A). The vaccine model was first subjected to AGGRESCAN for aggregation-prone regions. The residues having scores <0 are considered soluble while a positive value depicts the aggregations-prone residues as in Figure 7B. This step is followed by cabs-flex resulting in 10 models. The highest RSMF obtained was 7.117 angstrom for residue 282, and the lowest was 0.297 angstroms for residue 93 (Figure 7C).

### Disulfide engineering and *in-silico* cloning

**Figure 8:**
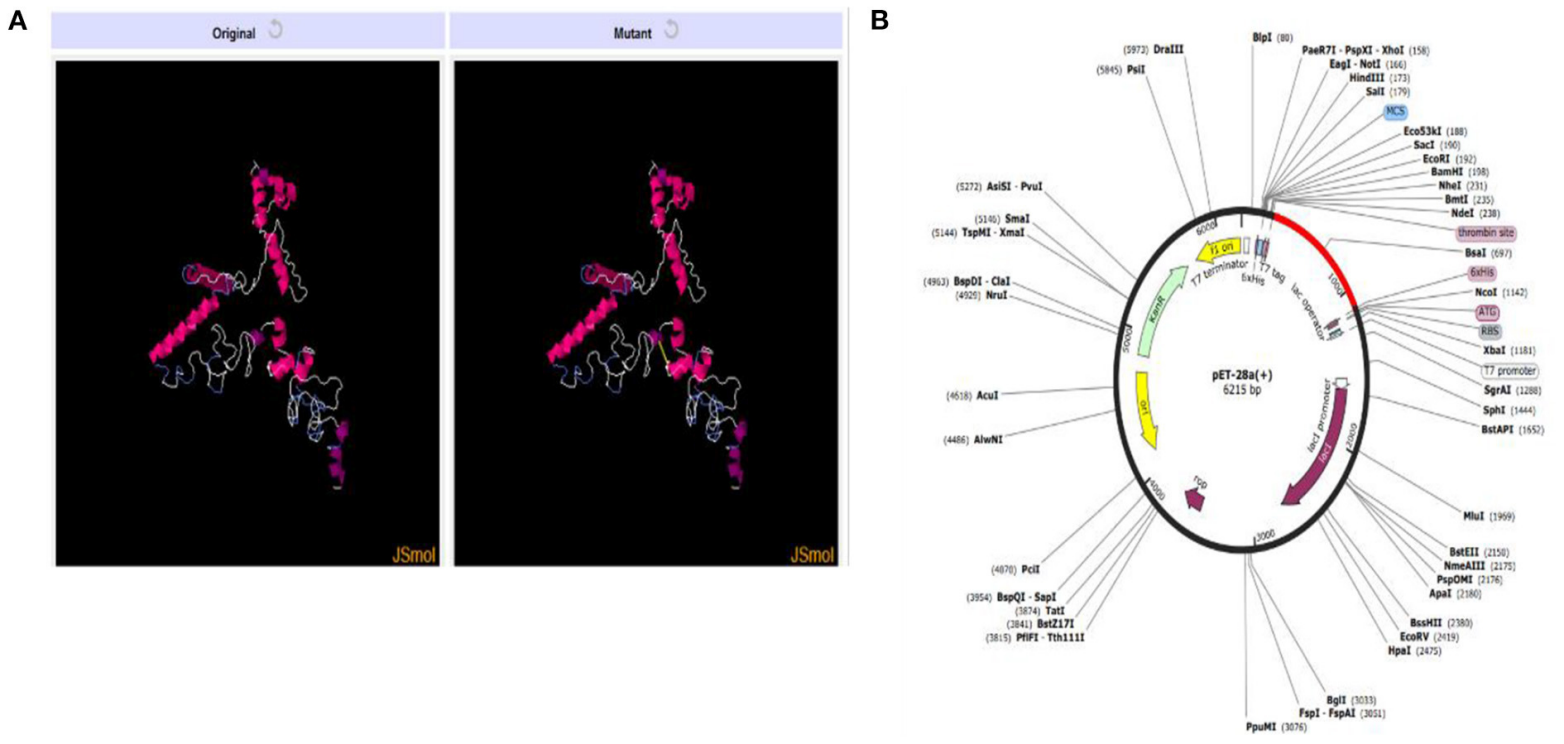
**(A)** Original and mutant vaccine model obtained by disulfide engineering. **(B)** Vaccine (in red) cloned in pET28a (+) vector.

The presence of disulfide bonds in any model confirms its structural stability. Disulfide engineering refers to marking the residues in the model as being considered unstable and mutating them as cysteine pairs. There were 18 cysteine pairs identified (Figure 8A). Mutated cysteine pairs included 3LYS-36THR (energy value 5.71 kcal/mol, X3 angle +121.45), 6PHE-12VAL (energy value 3.60 kcal/mol, X3 angle +115.46), 16SER-27THR (energy value 1.33 kcal/mol, X3 angle +96.65), 105LYS-108VAL (energy value 3.11 kcal/mol, X3 angle +95.23), 109TRP-112LYS (energy value 2.7 kcal/mol, X3 angle +119.05), 130ALA-140PRO (energy value 4.85 kcal/mol, X3 angle +88.29), 160ASP-163GLU (energy value 2.57 kcal/mol, X3 angle +107.9), 168PRO-182PRO (energy value 4.5 kcal/mol, X3 angle +103.79), 173ALA-178ALA (energy value 1.41 kcal/mol, X3 angle +85.64), 186LYS-192THR (energy value 5.21 kcal/mol, X3 angle +89.68), 195GLY-216THR (energy value 1.92 kcal/mol, X3 angle −107.05), 206SER-256GLN (energy value 3.21 kcal/mol, X3 angle +98.68), 208ALA-215ALA (energy value 6.31 kcal/mol, X3 angle −64.05), 218LYS-227GLY (energy value 2.89 kcal/mol, X3 angle −70.65), 238PRO-243GLU (energy value 2.93 kcal/mol, X3 angle −103.67), 245GLY-255GLY (energy value 5.57 kcal/mol, X3 angle −79.03), 246THR-266PRO (energy value 5.16 kcal/mol, X3 angle +73.56), and 275SER-278SER (energy value 3.07 kcal/mol, X3 angle −91.98).

*In-silico cloning* for the vaccine construct was also performed following the codon adaptation in which the sequence for the vaccine construct was reverse-translated. The CAI value obtained for the sequence was 1.0 and the calculated GC content was 53.66%. The expression system was *E. coli* K12 and the vaccine sequence was expressed in expression vector pET-28a (+) colored red in Figure 8B. The reverse translated sequence being cloned in the vector was: ATGATCAAACTGAAATTCGGTGTTTTCTTCACCGTTCTGCTGTCTTCTGCTTACGCTCACGGTACCCCGCAGAACATCACCGACCTGTGCGCTGAATACCACAACACCCAGATCTACACCCTGAACGACAAAATCTTCTCTTACACCGAATCTCTGGCTGGTAAACGTGAAATGGCTATCATCACCTTCAAAAACGGTGCTATCTTCCAGGTTGAAGTTCCGGGTTCTCAGCACATCGACTCTCAGAAAAAAGCTATCGAACGTATGAAAGACACCCTGCGTATCGCTTACCTGACCGAAGCTAAAGTTGAAAAACTGTGCGTTTGGAACAACAAAACCCCGCACGCTATCGCTGCTATCTCTATGGCTAACGAAGCTGCTGCTAAAGCTATCAAAGGTGAATCTGCTGACAAAGGTCCGGGTCCGGGTTGGTCTCTGACCAAAAACGACAAAACCGGTCCGGGTCCGGGTTCTCGTGACGACACCGAACGTTCTCGTGGTCCGGGTCCGGGTAACGCTTCTGCTGGTGCTGCTCGTACCGGTCCGGGTCCGGGTAAAGTTGACTCTACCCCGACCTCTAACGGTCCGGGTCCGGGTTCTAACGCTCGTACCCTGTCTAACGCTGGTCCGGGTCCGGGTGCTGCTACCGCTAAAACCGCTCAGTCTGGTCCGGGTCCGGGTGCTGCTTCTTCTTCTGCTGAACCGCGTGGTCCGGGTCCGGGTTACGAAAAAGGTACCGGTTCTCGTCTGGGTCCGGGTCCGGGTCAGATCGACGTTACCGACGAAGACCGTGGTCCGGGTCCGGGTGGTAACAACGACGAATCTAACATCTCTTTCAAAGAAAAA.

### Computational immune simulation

**Figure 9:**
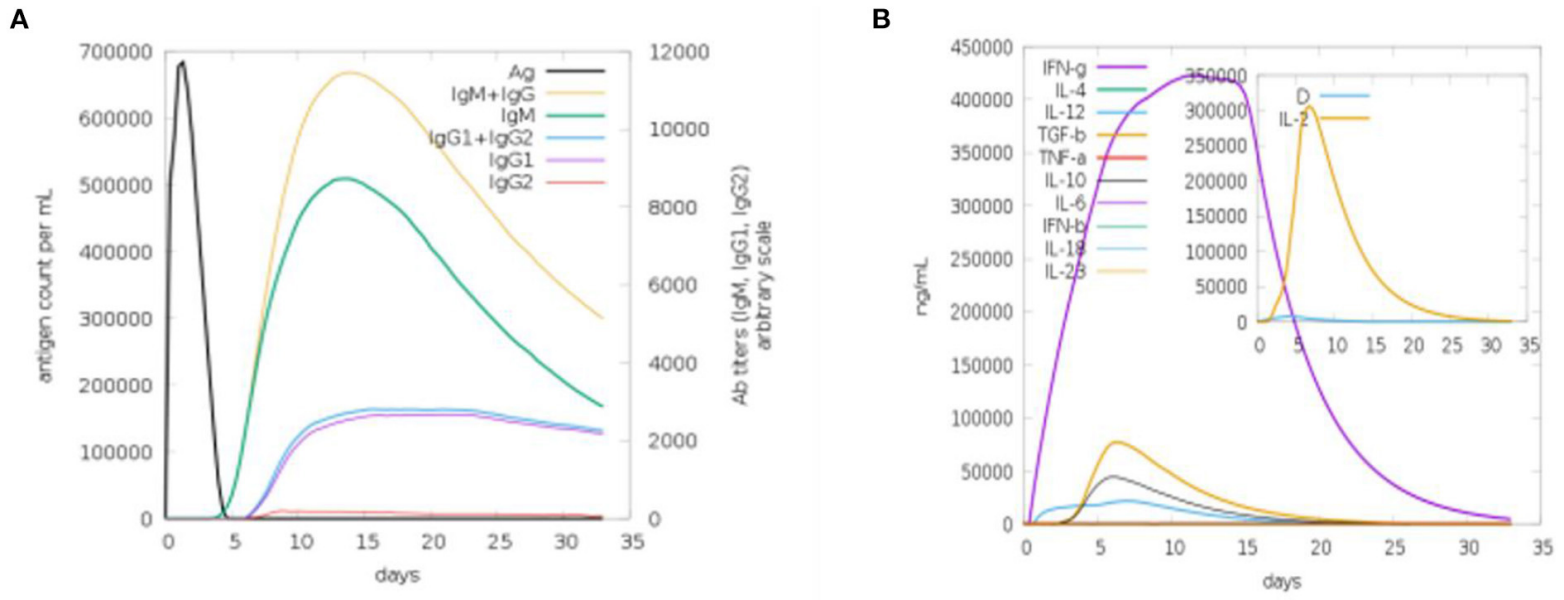
Computational immune simulation against the vaccine model. **(A)** Induction of immunoglobulins. **(B)** Induction of interleukins.

We used a C-ImmSim online webserver for the computational immune simulation demonstrating the immune responses expected against the designed multi-epitopes vaccine. Production of immunoglobulins and interleukins proved the effectiveness of the vaccine. Figure 9A shows that IgM production is 700,000 and IgM + IgG production is 10,000 in the first 15 days. Figure 9B is depicting the interleukins induction in response to the vaccine. The amount of IFN-g generated was >400,000 ng/ml. IFN-g levels are higher, indicating that the human immune system is activated (36).

### Molecular docking of vaccine

**Figure 10:**
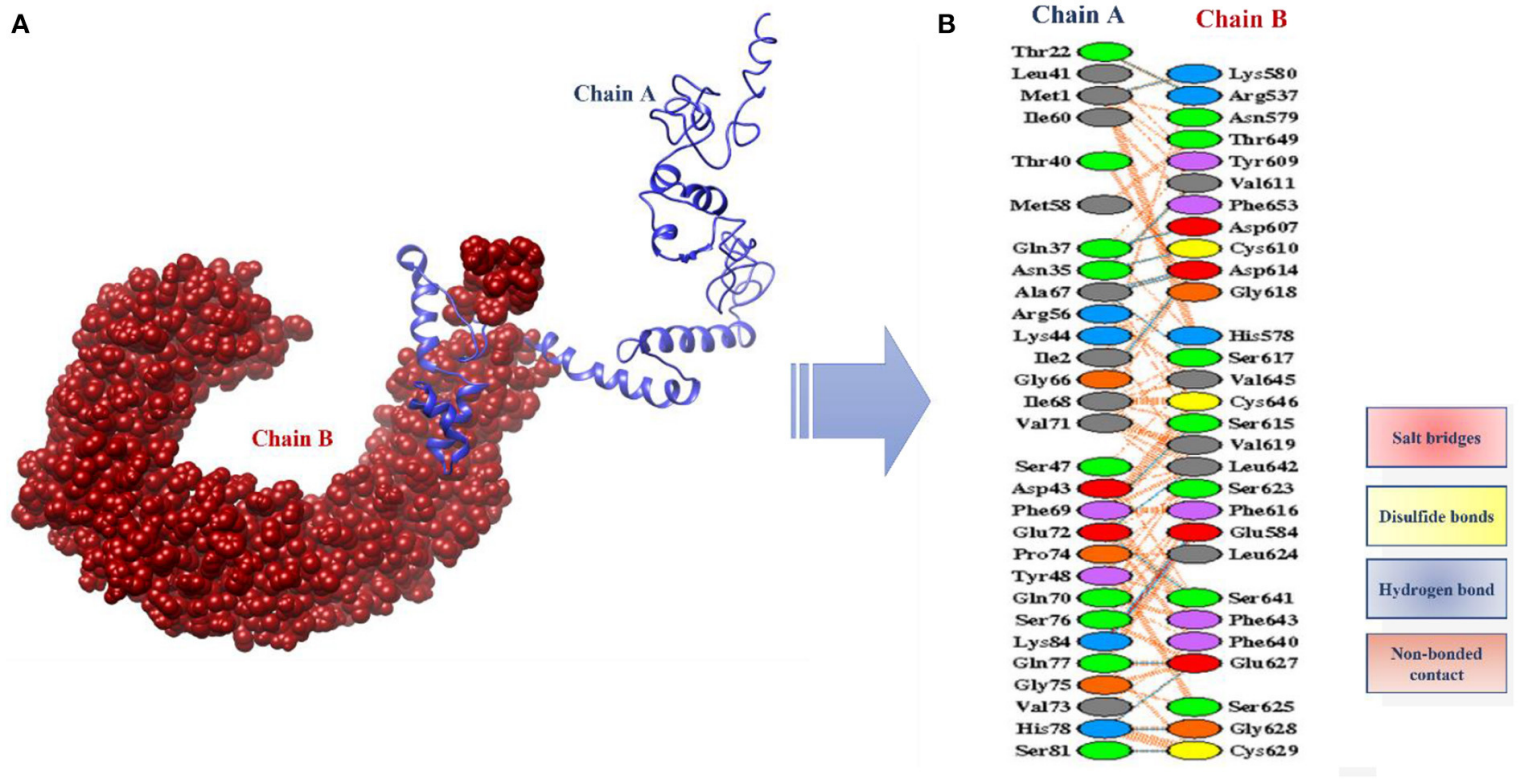
**(A)** Docked complex of vaccine model and TLR5 **(B)** Interactions between the chain A (vaccine) and the chain B (TLR5).

The designed vaccine was docked with the receptor TLR 5. TLR5 recognizes the flagellin of the bacterium and is highly expressed in patients infected with *B. pseudomallei* (37). TLRs are involved in inducing immunity and cytokine induction. Docking refers to the prediction of interactions between ligand (vaccine) and the receptor (TLR 5) (38). Here, the docked models were obtained from the ClusPro server and the top 10 are shown in Table 4. These models are arranged based on their cluster size. The clustering step was done using the pairwise IRMSD (39). The best docked complex obtained is shown in Figure 10A. The interactions between the vaccine (chain A) and TLR-5 (chain B) in a docked complex are presented in Figure 10B. The interactions in red color are salt bridges, while those in yellow are disulfide, and hydrogen bonds are in blue color. The non-bonded interactions are in orange.

**Table 4:** Models for the docked complexes of TLR5 and designed vaccine.

**Cluster**	**Members**	**Representative**	**Weighted score**
0	50	Center	−1,137.2
		Lowest energy	−1,137.2
1	36	Center	−965.5
		Lowest energy	−1,168.3
2	28	Center	−997.5
		Lowest energy	−1,150.1
3	23	Center	−1,127
		Lowest energy	−1,339.4
4	23	Center	−1,032.9
		Lowest energy	−1,133
5	22	Center	−1,216.4
		Lowest energy	−1,234.3
6	19	Center	−1,109.5
		Lowest energy	−1,127.4
7	17	Center	−970.8
		Lowest energy	−1,204.7
8	17	Center	−1,173.4
		Lowest energy	−1,173.4
9	17	Center	−1,034.6
		Lowest energy	−1,139.9

Models are represented in order of their cluster size. Number of members depicting the number of neighboring structures. Weighted score is depicting the energy of the structure present at the center and the neighboring structure with lowest energy in a cluster.

### Molecular dynamics simulation analysis and binding free energies estimation

**Figure 11:**
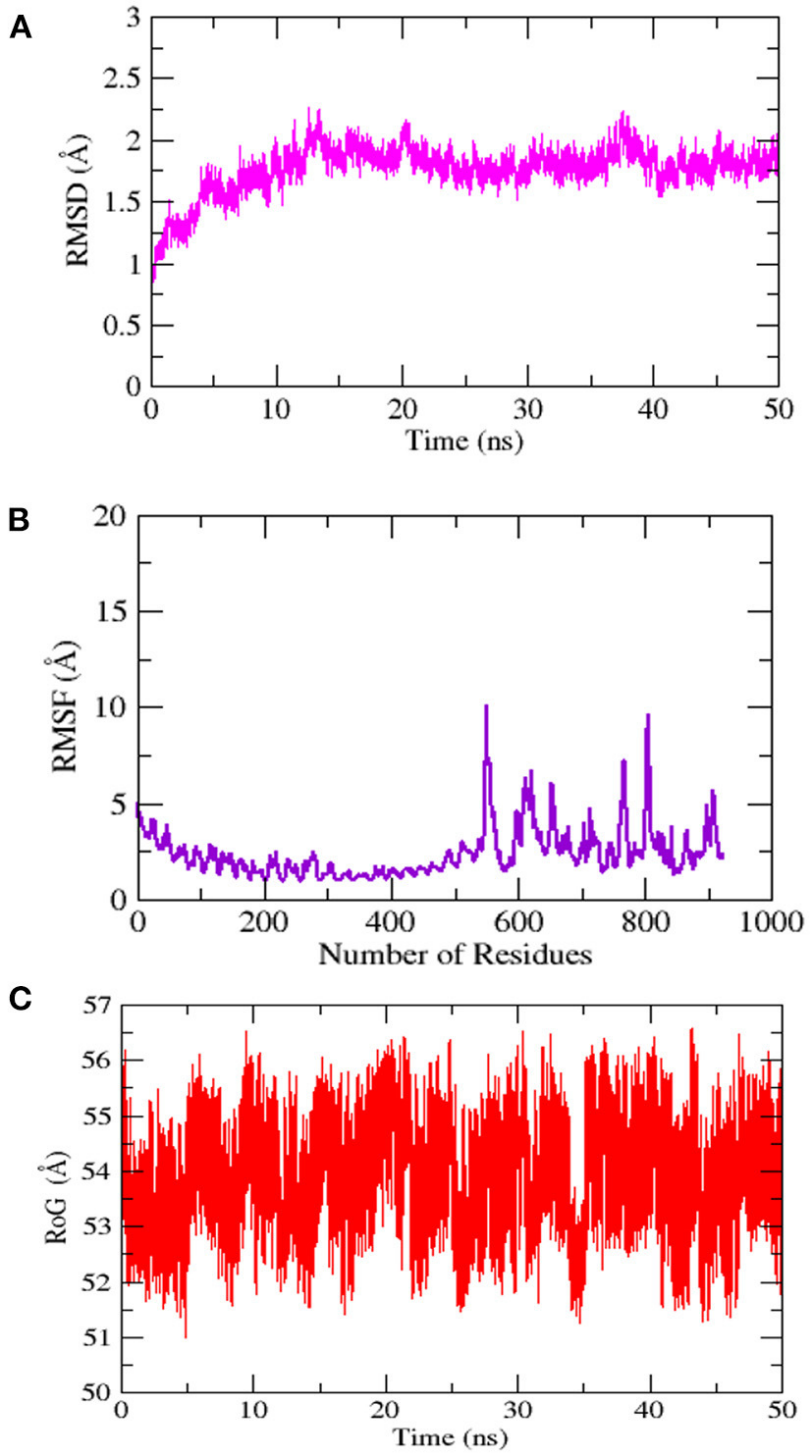
Simulation trajectories analysis **(A)** Root-mean-square deviation RMSD **(B)** Root-mean-square fluctuation (RMSF) and **(C)** Radius of gyration (RoG).

The stability and dynamic behavior of the docked Vaccine-TLR-5 complex was further assessed through molecular dynamics simulation analysis. The root-mean-square deviation (RMSD), root-mean-square fluctuation (RMSF), and radius of gyration (RoG) were computed for the system. RMSD analysis during simulation revealed that the complex reaches 7Å at 10.5 ns, however, this is due to the presence of loops. The graph became stable and showed maximum stability toward the end of simulation time as presented in Figure 11A. Next, in RMSF analysis the docked complex was analyzed to check the residue level fluctuations. In RMSF analysis, very low-level fluctuations were observed throughout the simulation time. The few fluctuations were due to the reason for vaccine adjustment at the proper docked site. However, these fluctuations did not affect the overall stability and binding mode of the vaccine to the receptor as shown in Figure 11B. Moreover, the intermolecular stability of the vaccine-TLR-5 can be also witnessed by the radius of gyration analysis, which predicted the maximum compactness of the complex as mentioned in Figure 11C. Additionally, the MM-GBSA analysis was utilized to estimate vaccine-TLR-5 complex free binding energies. The overall binding affinity of the vaccine-TLR-5 complex was a total of −168.35 kcal/mol. The net van der Waals and electrostatic energies are the most favorable in the docked complex formation. The gas phase energy of the complex dominates the overall energy of the system while polar energy is non-favorable in complex formation. The terms for different binding free energies are tabulated in Table 5.

**Table 5:** Binding free energies calculated for vaccine-TLR-5 complex.

**Energy Component**	**GB**	**PB**
VDWAALS	−2,327.5146	−2,327.5146
EEL	−24,524.7331	−24,524.7331
EGB	−5,713.6747	−5,548.8917
ESURF	159.6436	110.8892
G gas	−26,852.2478	−26,852.2478
G solv	−5,554.0311	−5,438.0024
TOTAL	−32,406.2788	−32,290.2502
**Receptor**		
VDWAALS	−2,294.5732	−2,294.5732
EEL	−24,479.7925	−24,479.7925
EGB	−5,727.4367	−5,564.9699
ESURF	159.4314	110.6366
G gas	−26,774.3657	−26,774.3657
G solv	−5,568.0052	−5,454.3333
**Vaccine construct**		
VDWAALS	−6.8361	−6.8361
EEL	−29.9481	−29.9481
EGB	−11.9397	−12.0813
ESURF	3.1747	2.6863
Gas	−191.0979	−36.7841
Solv	22.7391	−9.3949
TOTAL	−45.5491	−46.1791
**Differences (complex-Receptor-Vaccine construct)**		
VDWAALS	−126.1053	−126.1053
EEL	−64.9926	−64.9926
EGB	25.7016	28.1595
ESURF	−2.9626	−2.4337
Delta G sol	−191.0979	−191.0979
Delta Solv	22.7391	25.7258
DELTA Total	−168.3588	−216.8237

## Discussion

*B. pseudomallei* is a gram-negative bacterium that causes melioidosis and can be lethal if not adequately treated. It can sometimes act as a facultative intracellular pathogen, emphasizing the pathogenesis of the infection (40). Long medications can be used to treat the infection; however, reports have shown that the bacterium has developed resistance to several antibiotics. Many vaccines against this infection were designed and some of them are in preclinical trials as well. Here, we applied cost-effective immunoinformatic and reverse vaccinology approaches to design a multi-epitope vaccine against *B. pseudomallei*. Immunoinformatic approaches for vaccine designing can develop effective vaccines in less time and can provoke both innate and humoral immune responses (40). The previous vaccine development techniques are considered less capable as compared to multi-epitope vaccines (41). Core proteome was retrieved from 91 *B. pseudomallei* strains and subjected to various filters resulting in potential vaccine candidates. This was done *via* a subtractive proteomics approach in which desired proteins were filtered (42, 43). The extracellular, periplasmic, and outer membrane protein sequences were used to design a multi-epitope vaccine. These are the surface proteins involved in bacterial pathogenesis and are very crucial in the development of vaccines (42, 43). These proteins also play a role in pathogen attachment, cell entrance, and disease prognosis (44). The virulence of a protein is very important for disease development (45). These proteins were checked for their capability to be a part of the vaccine and went through various filters like allergenicity, antigenicity, adhesion probability, and solubility. The shortlisted vaccine proteins were used in epitope mapping. B-cell derived T-cell epitopes were used in vaccine construct as B- and T-cells play vital roles in inducing cell-mediated immunity against the pathogen (46). Predicted epitopes were prioritized and shortlisted for vaccine designing. Similar studies were also conducted against *Acinetobacter baumannii* (18), *Enterobacter hormaechei* (16), and *Enterobacter xiangfangensis* (17) etc. These studies prioritized several vaccine targets against the pathogens and proposed chimeric epitope peptides that can better induce humoral and cellular immunity.

The vaccine construct was designed by linking the epitopes with GPGPG linkers and joining the epitope to the adjuvant *via* the EAAAK linker. The purpose of utilizing linkers is to facilitate the separation of epitopes and adjuvants while avoiding overlaps (46, 47). Four different adjuvants were utilized, and the final construct with adjuvant CTB was 3D modeled and subjected to further analysis. CTB is a non-toxic part of Cholera toxin which, when linked to antigen, results in boosting the immunity and immune responses (48). CTB is associated with the induction of CD4+ T-cell immune activation (49). The vaccine model was visualized through Chimera to identify the loop regions followed by loop modeling and refinement. Loop modeling is involved in predicting secondary structure elements for the loop. Loop regions play a crucial role in several biochemical functions (49, 50). The structure of the vaccine model was improved by refinement because some structures are not predicted accurately, hindering the protein from function properly, and causing difficulty in structure-based research (49, 50). Structural validation of the 3D model was done, and the Ramachandran plot (51) explained the stability of the structure having 92.6% of its residues in the most favored regions. Disulfide engineering and codon optimization were important steps performed to improve structural stability (52). The main aim of the codon optimization was to increase the vaccine's expression level resulting in its higher efficiency for experimental research (18). To generate proper immune responses, the designed vaccine construct should have the binding capability with immune cell receptors. Among immune cells receptors, the TLRs family plays a vital role in the generation of proper immune responses.

In this study, we performed a docking analysis to assess the vaccine's binding potency with TLR-5. The server generates 10 complexes with different binding energy scores. From the docking analysis, the designed vaccine can interact with TLR-5 molecules and can provoke proper immune reactions. For long-term immunity, the docking stability of the docked complexes must be maintained. To evaluate the dynamic movement of the docked complex, a molecular dynamics simulation approach was performed. The results of *in-silico* host immune simulation revealed that the chimeric vaccine can induce a proper immune response in the host body. The immune response was observed in the form of different antibodies and cytokines. According to our findings, the proposed vaccine is promising and capable of eliciting a proper immune response against *B. pseudomallei*.

## Conclusions

Antibiotic abuse in animals, humans, and agriculture has resulted in the emergence of antibiotic-resistant bacterial infections, which has significantly raised hospitalization, community mobility, and mortality rates. Melioidosis, a condition that can be fatal, is caused by *B. pseudomallei*. There is no licensed vaccination to protect against *B. pseudomallei* infection. Immunoinformatics, bioinformatics, and reverse vaccinology were employed to speed up vaccine target discovery while also lowering costs and saving time. In the current research, a multi-epitopes-based vaccine was designed that may produce innate and adaptive immunity against *B. pseudomallei* using a variety of bioinformatics, immunoinformatic, and reverse vaccinology methodologies. For the above-said purpose, the first proteins were shortlisted from pathogen core proteins and utilized for probable antigenic, non-allergic, non-toxic, and good water-soluble epitopes prediction. Several analyses demonstrate that the developed vaccine model is capable of inducing a proper innate and adaptive immune response against the target pathogen. However, wet laboratory confirmation and validations are strongly advised to validate *in-silico* findings. Although the *in-silico* results are encouraging, additional efforts are required, such as the use of more accurate servers/tools to validate the results.

## Data availability statement

Publicly available datasets were analyzed in this study. This data can be found here: NCBI GenBank.

## Author contributions

All authors listed have made a substantial, direct, and intellectual contribution to the work and approved it for publication.

## Conflict of interest

The authors declare that the research was conducted in the absence of any commercial or financial relationships that could be construed as a potential conflict of interest.

## Publisher's note

All claims expressed in this article are solely those of the authors and do not necessarily represent those of their affiliated organizations, or those of the publisher, the editors and the reviewers. Any product that may be evaluated in this article, or claim that may be made by its manufacturer, is not guaranteed or endorsed by the publisher.
